# Secondary fragility fractures after hip fracture surgery in four thousand, four hundred and eighteen older adults: risk factors and internal validation of an interpretable machine-learning model

**DOI:** 10.1007/s00264-026-07002-y

**Published:** 2026-09-07

**Authors:** Shizan He, Di Wu, Dajun Jiang, Sen Lin, Yuquan Bian, Huipeng Shi, Weitao Jia

**Affiliations:** 1 Department of Orthopaedics, Shanghai Sixth People’s Hospital Affiliated to Shanghai Jiao Tong University School of Medicine, Shanghai, 200233 China; 2 Postgraduate Training Base of Jinzhou Medical University at Shanghai Sixth People’s Hospital, Jinzhou Medical University, Jinzhou, 121001 China

**Keywords:** Hip fracture, Secondary fragility fracture, Risk factors, Machine learning, Gradient boosting

## Abstract

**Background:**

Secondary fragility fractures remain a major complication after hip fracture surgery in older adults, yet individualized postoperative risk stratification remains challenging. We aimed to identify factors associated with secondary fragility fracture and internally validate an interpretable machine-learning model for three year refracture risk.

**Methods:**

We retrospectively analyzed adults aged ≥ 65 years who underwent surgery for a first low-energy hip fracture between 2014 and 2021. Patients were followed for three years for secondary fragility fracture. Demographic, clinical, functional, and rehabilitation variables were extracted from electronic medical records. Cox regression with multiple imputation was performed to evaluate time-to-event associations. XGBoost was developed as the primary prediction model and compared with SVM and Cox regression. Model performance was assessed using a held-out test set and decision curve analysis.

**Results:**

Among 4,418 patients, 594 (13.4%) sustained a secondary fragility fracture. Increased refracture risk was associated with older age, osteoporosis, low BMI, female sex, cardiovascular disease, visual impairment, syncope, non-standard rehabilitation, and postoperative Harris hip score < 80. In the test set, XGBoost achieved an AUC of 0.701 (95% CI, 0.657–0.743), with 61.8% sensitivity and 67.2% specificity at the default threshold. Decision curve analysis demonstrated the highest net benefit for XGBoost across threshold probabilities of approximately 7%–32%.

**Conclusions:**

Secondary fragility fractures were associated with skeletal fragility, fall-related vulnerability, and impaired postoperative recovery. The interpretable XGBoost model showed moderate discrimination and potential clinical utility as an adjunctive screening tool to identify patients who may benefit from intensified secondary prevention. External validation and prospective evaluation are required before routine clinical implementation.

**Graphical abstract:**

**Figure:**
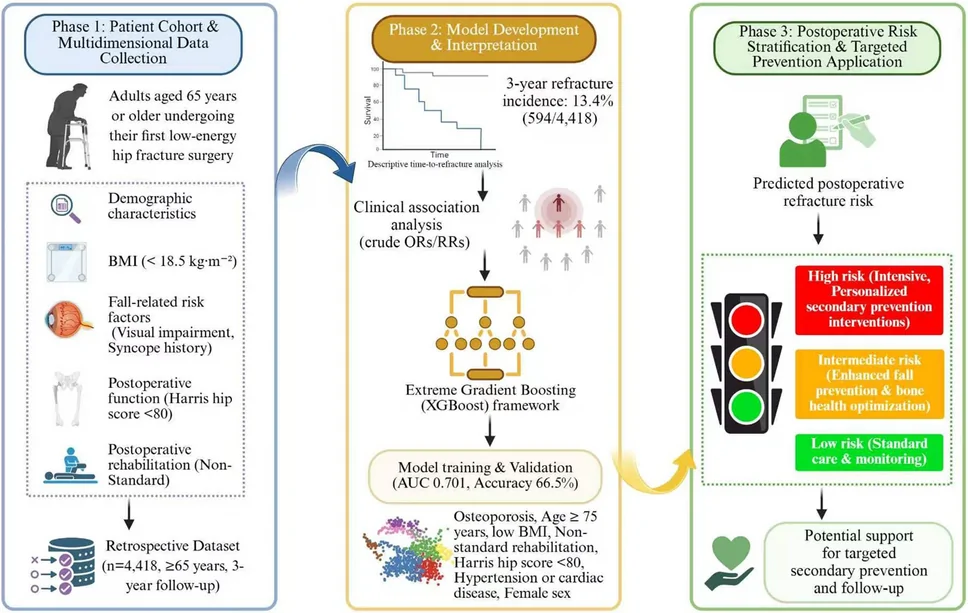

## Introduction

Hip fractures are among the most devastating and resource-intensive osteoporotic injuries in older adults and remain a major challenge to ageing health-care systems worldwide [1–3]. Despite advances in surgical timing, anaesthetic management, perioperative care, and rehabilitation, the long-term prognosis after hip fracture remains poor, with persistent functional dependence, recurrent falls and fractures, institutionalisation, and excess mortality [4–7]. These outcomes highlight that successful fixation or arthroplasty addresses only the acute structural injury; the underlying skeletal fragility, frailty, and fall susceptibility frequently persist after surgery and continue to place patients at substantial risk of further adverse events.

Secondary fragility fracture is particularly important because a first hip fracture marks a period of heightened vulnerability to subsequent osteoporotic injury. Previous cohort studies have reported that approximately 10%–30% of patients sustain another fragility fracture within several years after hip fracture surgery, commonly involving the contralateral hip, vertebrae, distal radius, or proximal humerus [8–11]. Refracture risk is multifactorial and extends beyond osteoporosis alone. Advanced age, reduced bone mineral density, low body mass, visual impairment, recurrent falls, sarcopaenia, medical comorbidity, and inadequate osteoporosis treatment have all been implicated, while postoperative functional recovery and rehabilitation may further modify the risk trajectory [12–14]. Thus, clinically meaningful secondary prevention requires assessment of both skeletal vulnerability and the broader functional and fall-related context in which refractures occur.

Risk prediction after hip fracture, however, remains challenging. Conventional statistical approaches are valuable for estimating associations and maintaining interpretability, but prediction based on routinely collected clinical data may be limited when multiple interacting domains, including skeletal health, comorbidity, sensory impairment, functional recovery, and rehabilitation, are considered simultaneously [15, 16]. Machine-learning approaches offer a complementary framework for modelling complex and potentially nonlinear relationships, but increasing algorithmic complexity does not necessarily translate into superior or clinically useful prediction. [17, 18]. Recent studies illustrate both the promise and limitations of these approaches. Kim et al. developed a CT-based deep-learning model for subsequent fracture prediction after hip fracture and reported AUCs of approximately 0.73–0.74 at two, three and five year horizons [16]. Shimizu et al. applied several machine-learning algorithms to patients with surgically treated fragility fractures and found moderate predictive performance, with rheumatoid arthritis and chronic kidney disease emerging as important features [19]. In a substantially larger cohort of 74,396 patients from the Dutch Hip Fracture Audit, Dijkstra et al. found that conventional logistic regression performed comparably to more complex machine-learning approaches for mortality prediction and was favoured because of its interpretability [20]. Together, these studies indicate that the clinical value of predictive modelling depends not only on discrimination, but also on transparent validation, interpretability, calibration, and whether predicted risk can be translated into meaningful clinical action.

Several gaps therefore remain in the prediction of secondary fragility fracture after hip fracture surgery. Existing studies have often focused on a single fracture site, imaging-derived skeletal features, or heterogeneous fragility-fracture populations, whereas postoperative functional recovery, rehabilitation quality, visual impairment, and syncope-related fall susceptibility have been less consistently incorporated into integrated prediction frameworks [21, 22]. Moreover, relatively few studies have evaluated whether a machine-learning model provides potential clinical benefit across realistic decision thresholds rather than relying on discrimination alone. These limitations are especially relevant in secondary fracture prevention, where the objective is not to establish a diagnosis but to identify patients who may warrant intensified multidisciplinary assessment and follow-up.

Accordingly, we conducted a large retrospective cohort study of older adults undergoing surgery for a first fragility hip fracture at a high-volume tertiary orthopaedic centre. We first characterised the demographic, skeletal, comorbidity, fall-related, rehabilitation, and functional factors associated with secondary fragility fracture during three years of follow-up and incorporated Cox proportional hazards modelling to account for the time-to-event nature of refracture. We then developed and internally validated an interpretable extreme gradient boosting (XGBoost) model and compared its predictive performance with conventional comparators [23]. In addition to discrimination, we evaluated decision-analytic net benefit to better define the potential clinical role of the model. Rather than seeking to replace established fracture-risk assessment or clinical judgement, our objective was to determine whether routinely available multidimensional clinical information could support postoperative screening and triage for intensified secondary prevention in older adults after hip fracture surgery.

## Methods

### Study design and setting

We conducted a retrospective cohort study at Shanghai Sixth People’s Hospital (National Orthopedic Medical Center), a high-volume tertiary referral centre specialising in geriatric trauma and hip reconstruction. Consecutive older adults who underwent surgery for a first hip fracture and were successfully discharged between January 2014 and December 2021 were identified from the institutional electronic medical record system and orthopaedic registry. The study was designed and reported in accordance with STROBE and TRIPOD recommendations for prognostic research. The study protocol was approved by the Ethics Committee of Shanghai Sixth People’s Hospital (approval number: 2025-KY-42). Written informed consent was waived owing to the retrospective design and the use of de-identified data. All procedures were conducted in accordance with the principles of the Declaration of Helsinki.

### Participants

Eligible patients met all of the following criteria: (1) Age ≥ 65 years at the time of admission; (2) First low energy fragility hip fracture involving the femoral neck or intertrochanteric region, confirmed by radiography; (3) Treatment with surgical fixation or arthroplasty within the same admission; (4) Pre-fracture community-dwelling status or able to ambulate with or without aids; (5) Available baseline clinical data and at least one postoperative functional assessment. We excluded patients with: (1) High energy trauma, pathological fractures due to malignancy, or periprosthetic fractures; (2) Metabolic bone disease other than primary osteoporosis (for example severe renal osteodystrophy, Paget disease, osteomalacia due to rare inherited disorders); (3) Multiple major injuries that precluded standard hip rehabilitation pathways; (4) Documented severe cognitive impairment precluding reliable functional evaluation, without a proxy informant; (5) Incomplete follow-up for refracture status or death, despite attempts to contact patients, relatives and primary care providers. If patients experienced more than one hip fracture during the study period, only the first qualifying event was considered as the index hip fracture; subsequent events contributed to the refracture outcome.

### Outcome definition and follow-up

The primary outcome was the first new fragility fracture occurring within three years after index hip fracture surgery. Refracture was defined as a low energy fracture involving classical osteoporotic sites, including contralateral hip, clinical or radiographic vertebral fractures, distal radius and proximal humerus, confirmed by imaging and clinical documentation. Traumatic fractures from high energy mechanisms and fractures of the skull, facial bones, hands or feet were not counted as refractures.

Follow-up was scheduled at six, 12, 24, and 36 months after surgery. Refracture and vital status were ascertained using complementary sources, including scheduled outpatient visits, inpatient and outpatient records, regional health information exchange data, and structured telephone interviews conducted by trained research nurses. For patients who moved outside the region, outcomes were obtained from patients or family members and, whenever possible, verified using local hospital records or imaging reports. Deaths were confirmed through institutional or official records. Patients with a confirmed refracture within 36 months were classified as having the primary outcome. Patients without refracture were included only if refracture-free status was confirmed through 36 months, whereas those who died or remained lost to follow-up before 36 months without a documented refracture were excluded after all available record-linkage and contact attempts had been exhausted.

### Clinical and rehabilitation variables

We extracted demographic, clinical, surgical and rehabilitation variables that were a priori considered relevant to refracture risk. Baseline covariates included age, sex, body mass index, type of index fracture (femoral neck vs intertrochanteric). Comorbidity data included hypertension or cardiac disease, cerebrovascular disease, visual impairment, and a history of syncope. Osteoporosis was defined by a DXA T-score ≤ − 2.5 when available or by a documented clinical diagnosis; the index hip fracture alone was not used for classification. Osteoporosis medication data were incompletely recorded and were therefore excluded from model development but acknowledged as a potential source of residual confounding.

We paid particular attention to fall-related and neuromuscular factors. Visual impairment was defined as corrected visual acuity worse than 0.3 in the better eye or documented diagnosis of cataract, macular degeneration or other conditions severely limiting vision. Hearing impairment, a history of recurrent falls within the preceding year and a history of unexplained syncope were obtained from admission notes, geriatric assessments and cardiology records. Cognitive status and delirium during hospitalisation were recorded based on documented diagnoses or standardised screening, where available.

Functional recovery was evaluated using the Harris hip score at standardised time points after surgery. For the present analysis, a Harris score < 80 points at the available postoperative assessment was used to denote poor functional recovery. Rehabilitation quality was classified as standard when patients completed a structured, therapist-supervised hip fracture rehabilitation programme (≥ 3 sessions/week for ≥ 12 consecutive weeks) including strengthening, gait and balance, mobility, and functional training. Rehabilitation was classified as non-standard if any criterion was not met, including shorter duration, lower supervision frequency, home exercise alone, premature discontinuation, or absence of a documented structured programme. Where available, reasons for non-standard rehabilitation were also recorded. Receipt and persistence of osteoporosis medications during follow-up were also captured but not used as primary predictors in the baseline model.

### Data preprocessing

Bone mineral density (BMD) T-scores were recorded when available from dual-energy X-ray absorptiometry (DXA) performed before or after surgery. FRAX ten year probability scores were not systematically calculated in this retrospective cohort. Detailed fall histories (frequency, circumstances, and environmental hazards) and complete longitudinal medication records (e.g., bisphosphonate persistence, vitamin D levels) were incompletely captured in the electronic medical record and were not used as primary predictors.

Data were checked for consistency and outliers. Continuous variables were inspected visually and, where appropriate, truncated at extreme percentiles to reduce the influence of implausible values. For the conventional regression analysis, we created binary indicators for age ≥ 75 years, Harris score < 80 and presence of osteoporosis, visual impairment and syncope history, in line with clinical decision thresholds. Missing-data handling differed according to the analytic purpose. For Cox regression, missing predictor values were handled using multiple imputation by chained Eqs. (20 imputations) under a missing-at-random assumption, with pooled estimates obtained using Rubin’s rules. For machine-learning analyses, imputation was incorporated within the training pipeline to prevent information leakage: median values for continuous variables and mode values for categorical variables were estimated from the training set and subsequently applied unchanged to the held-out test set. Outcomes were not imputed.

### Conventional statistical analysis

Baseline characteristics were summarised as means with standard deviations or medians with interquartile ranges for continuous variables and as counts with percentages for categorical variables. Between-group comparisons were performed using Student’s t test or the Mann–Whitney U test for continuous variables and the χ^2^ test or Fisher’s exact test for categorical variables, as appropriate. For descriptive assessment of associations with three year refracture status, crude odds ratios (ORs) and relative risks (RRs), together with 95% confidence intervals, were calculated directly from the refracture and non-refracture groups.

Because secondary fragility fracture is a time-to-event outcome, a Cox proportional hazards model incorporating the same candidate clinical predictors was additionally fitted to account for the timing of the first refracture and to provide a conventional time-to-event comparator for the predictive analyses. The proportional hazards assumption was evaluated using Schoenfeld residuals. Among patients who experienced refracture, Kaplan–Meier was used descriptively to characterize time to refracture. To examine clinically relevant differences, and numbers at risk were displayed below the survival curves. All tests were two-tailed, and a *P*-value < 0.05 was considered statistically significant.

### Machine-learning model development

To develop a predictive model for 3-year refracture, we constructed a machine-learning pipeline using extreme gradient boosting (XGBoost) as the primary algorithm. A support vector machine (SVM) with a radial basis function (RBF) kernel was employed as a baseline comparator. The cohort was randomly split into a training set (70%) and an independent test set (30%) with stratification by outcome to preserve the event rate. Candidate predictors included routinely documented demographic, clinical, rehabilitation, and postoperative functional variables available during postoperative follow-up, with the aim of supporting postoperative risk stratification. For the SVM model, continuous variables were standardized using Z-score normalization (mean = 0, standard deviation = 1) to prevent features with larger scales from dominating the objective function. For the XGBoost model, raw features were used because tree-based algorithms are inherently invariant to monotonic feature scaling.

To optimize model performance and prevent overfitting, we implemented a Grid Search strategy combined with fivefold stratified cross-validation on the training set. The dataset was divided into five folds; in each iteration, the model was trained on four folds and validated on the fifth. This process was repeated for various hyperparameter combinations (including *learning rate*, *max depth*, *gamma*, and *subsample rate*) to identify the optimal configuration. The primary optimization objective was to maximize the area under the receiver operating characteristic curve (AUC).

### Model evaluation and internal validation

The final models, trained with the optimal hyperparameters, were evaluated on the held-out test set. Model discrimination was assessed using receiver operating characteristic curves and AUC. To evaluate the statistical robustness of the AUC estimates, 95% CIs for the AUC were calculated using the bootstrap method with 1,000 resamples. In addition to AUC, accuracy, sensitivity, specificity, and F1 score were calculated to provide a comprehensive performance profile. To evaluate the potential clinical utility of the prediction models beyond discrimination, decision curve analysis was performed across a range of threshold probabilities. Net benefit was calculated for XGBoost, SVM, and Cox regression and compared with the treat-all and treat-none strategies.

### Model interpretability

To enhance the clinical utility and transparency of the machine-learning model, we analyzed the contribution of each variable to the prediction refracture risk. Unlike "black-box" approaches, XGBoost provides an intrinsic mechanism to quantify feature importance. The importance of each clinical characteristic was calculated based on its contribution to the construction of the boosted decision trees. Specifically, the feature importance score reflects the relative utility of each variable in improving the model's predictive accuracy. Features with higher scores indicate a stronger influence on the model’s decision-making process regarding the patient's risk group.We ranked all input variables by their importance scores to identify the top predictors associated with the outcome.

### Software

All data analyses were performed using Python (Version 3.9) with the Pandas, SciPy, and Lifelines libraries. The machine-learning pipelines were implemented using the *Scikit-learn* library for data processing and SVM models, and the *XGBoost* library for gradient boosting models. Data visualization was conducted using *Matplotlib* and *Seaborn*.

## Results

### Study population and incidence of refracture

**Fig. 1:**
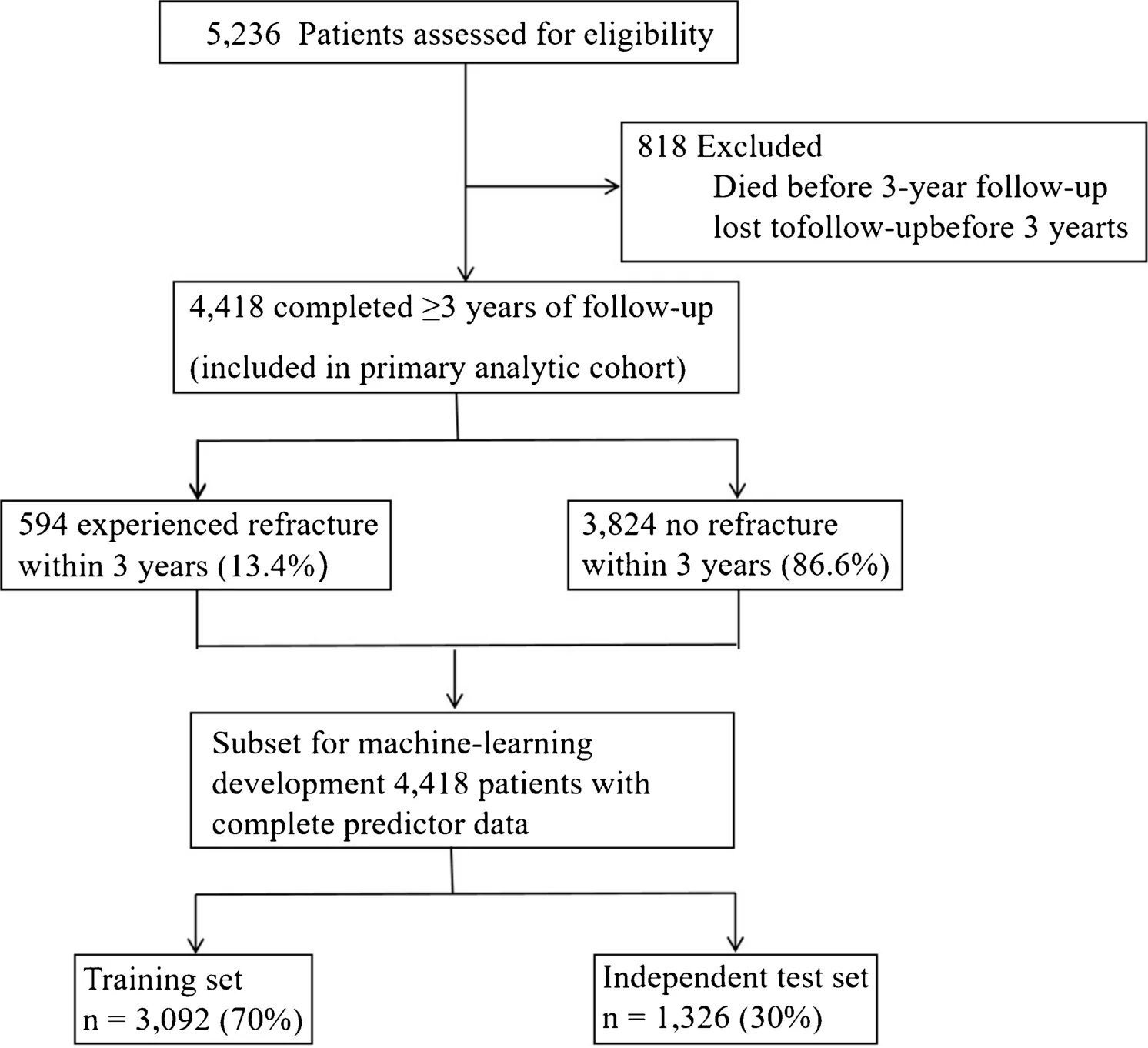
Flow diagram of study participants

During the study period, a total of 5,236 older adults underwent surgery for a first fragility hip fracture at our centre after applying the predefined inclusion and exclusion criteria. After exclusion of patients who died or were lost to follow-up before three years, 4,418 patients (84.4%) completed at least three years of follow-up and formed the primary analytic cohort (Fig. 1). The mean age was 76.5 ± 8.4 years (range, 65–95); 2,733 patients (61.9%) were 75 years of age or older. Women accounted for 2,728 patients (61.7%). Among 818 excluded patients (lost to follow-up or died before 3 years), the mean age was 79.2 ± 9.1 years, significantly older than in the analytic cohort (t = 8.05, *P* < 0.001), whereas the sex distribution was similar (χ^2^ = 1.23, *P* = 0.269).

Baseline characteristics stratified by refracture status are shown in Table 1. Compared with patients without refracture (*n* = 3,824), those with refracture (*n* = 594) were more likely to be 75 years of age or older (74.9% vs 59.8%), female (69.2% vs 60.6%), and underweight (BMI < 18.5 kg/m^2^, 22.4% vs 12.9%) (all *P* < 0.001). Osteoporosis was more common in the refracture group (74.4% vs 50.6%, *P* < 0.001), as were hypertension or cardiac disease (68.0% vs 61.1%, *P* = 0.001). Visual impairment (25.1% vs 18.1%, *P* < 0.001) and a history of syncope (12.8% vs 9.6%, *P* = 0.019) were also more frequent among patients with refracture. Intertrochanteric fracture patterns were similar between groups (56.2% vs 54.3%, *P* = 0.415). Postoperatively, non-standard rehabilitation (37.9% vs 27.9%, *P* < 0.001) and a Harris hip score below 80 (43.1% vs 33.2%, *P* < 0.001) were more common among patients who subsequently sustained refracture.

**Table 1:** Univariable analysis of factors associated with postoperative refracture in older adults with hip fracture

Variable	Refracture group (*n* = 594)	Control group (*n* = 3,824)	χ^2^ value	*P* value
Demographic characteristics				
Age ≥ 75 years	445 (74.9)	2,288 (59.8)	48.94	< 0.001
Female sex	411 (69.2)	2,317 (60.6)	15.74	< 0.001
BMI < 18.5 kg·m⁻^2^	133 (22.4)	493 (12.9)	37.36	< 0.001
Bone health and comorbidities				
Osteoporosis	442 (74.4)	1,936 (50.6)	116.06	< 0.001
Hypertension or cardiac disease	404 (68.0)	2,335 (61.1)	10.25	0.001
Cerebrovascular disease	104 (17.5)	549 (14.4)	3.81	0.051
Fall-related risk factors				
Visual impairment	149 (25.1)	692 (18.1)	15.84	< 0.001
History of syncope	76 (12.8)	367 (9.6)	5.48	0.019
Fracture and surgical characteristics				
Intertrochanteric fracture	334 (56.2)	2,078 (54.3)	0.67	0.415
Postoperative rehabilitation and function				
Non-standard rehabilitation	225 (37.9)	1,066 (27.9)	24.39	< 0.001
Harris hip score < 80	256 (43.1)	1,270 (33.2)	21.79	< 0.001

Data are presented as number (percentage); *BMI* body mass index

Continuous DXA T-scores were available for 442 patients (10.0%), whereas FRAX probabilities were not systematically available. The availability and missingness of additional candidate predictors, including recurrent-fall history, osteoporosis medication use, medication persistence, and serum 25-hydroxyvitamin D, are summarized in Supplementary Table S1.

### Incidence and timing of secondary fragility fractures

**Fig. 2:**
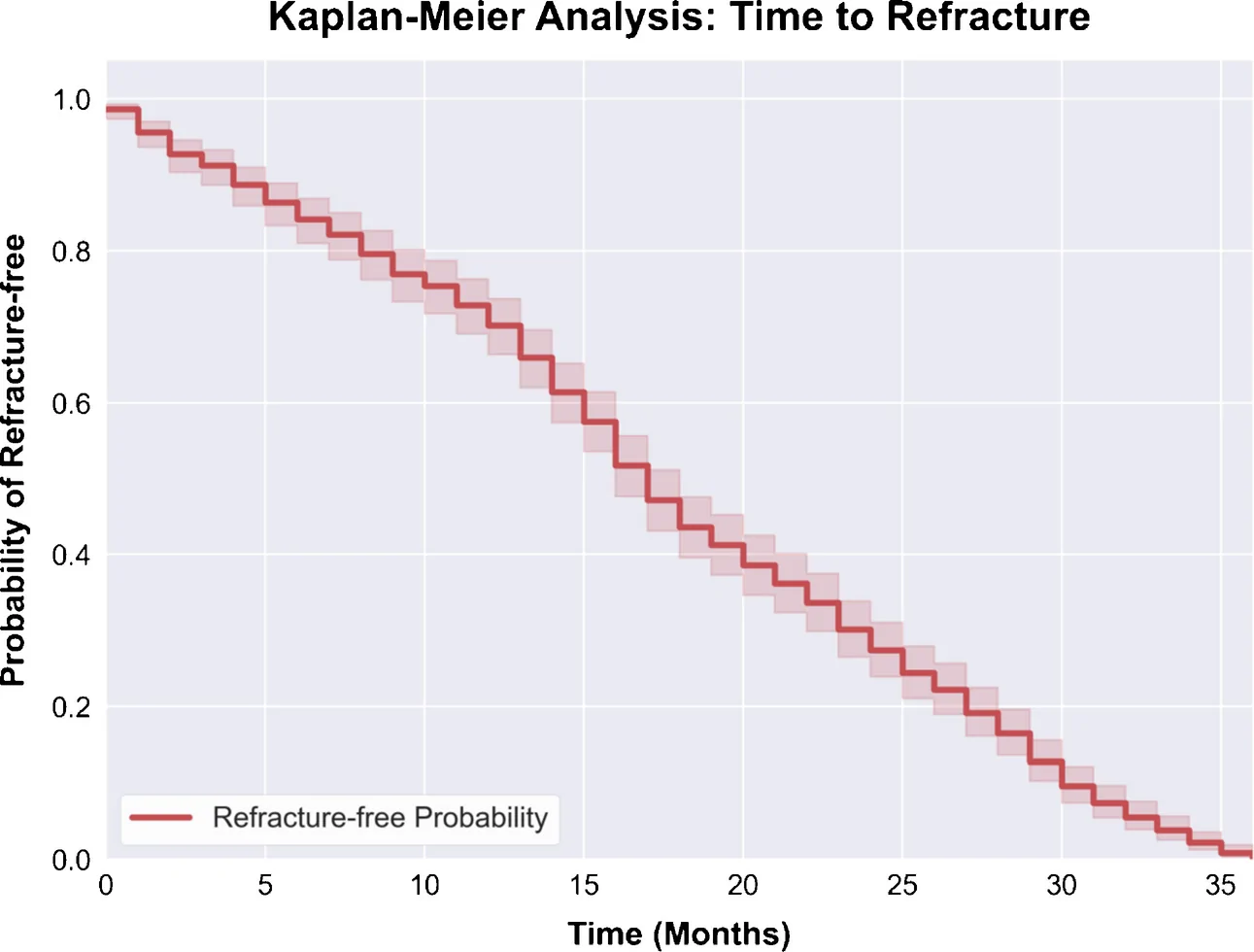
Kaplan–Meier distribution of time to secondary fragility fracture among the 594 patients who experienced refracture within 3 years

During the three year follow-up, 594 of 4,418 patients (13.4%) sustained a secondary fragility fracture, most commonly involving the contralateral hip or vertebrae. Among these 594 patients with refracture, the Kaplan–Meier curve of time from index surgery to refracture showed a steady decline in the proportion remaining fracture-free, with approximately 72% not yet refractured at 12 months and 29% at 24 months. The median time to refracture in this case group was 19.4 months (95% confidence interval, 17.2 to 21.6), indicating that a substantial proportion of secondary fragility fractures occurred during the second postoperative year (Fig. 2). Stratified analyses among these refracture cases showed differences in the timing of refracture according to osteoporosis status, rehabilitation status, and postoperative Harris hip score (Figure S1). These findings suggest that both skeletal fragility and postoperative functional recovery were associated with the risk and timing of secondary fragility fractures.

### Risk factors for postoperative refracture

Crude association estimates are presented in Table 2. Osteoporosis showed the largest association with three year refracture status (OR, 2.84; 95% CI, 2.33–3.45; RR, 1.47; 95% CI, 1.39–1.56; *P* < 0.001). Age ≥ 75 years was associated with higher refracture occurrence (OR, 2.00; 95% CI, 1.65–2.44; RR, 1.25; 95% CI, 1.19–1.32; *P* < 0.001), as was BMI < 18.5 kg/m^2^ (OR, 1.95; 95% CI, 1.57–2.42; RR, 1.74; 95% CI, 1.46–2.06; *P* < 0.001).

**Table 2:** Crude association estimates for factors associated with secondary fragility fracture within 3 years after hip fracture surgery in older adults

Variable	OR (95% CI)	RR (95% CI)	P value
Age ≥ 75 years	2.00 (1.65–2.44)	1.25 (1.19–1.32)	< 0.001
Female sex	1.46 (1.21–1.76)	1.14 (1.08–1.21)	< 0.001
BMI < 18.5 kg·m⁻^2^	1.95 (1.57–2.42)	1.74 (1.46–2.06)	< 0.001
Osteoporosis	2.84 (2.33–3.45)	1.47 (1.39–1.56)	< 0.001
Hypertension or cardiac disease	1.36 (1.13–1.63)	1.11 (1.05–1.18)	0.001
Cerebrovascular disease	1.27 (1.01–1.59)	1.22 (1.01–1.48)	0.051
Visual impairment	1.52 (1.24–1.86)	1.39 (1.19–1.62)	< 0.001
History of syncope	1.38 (1.06–1.80)	1.33 (1.06–1.68)	0.019
Intertrochanteric fracture as index fracture	1.08 (0.91–1.28)	1.03 (0.96–1.12)	0.415
Non-standard rehabilitation	1.58 (1.32–1.89)	1.36 (1.21–1.52)	< 0.001
Harris hip score < 80	1.52 (1.28–1.82)	1.30 (1.17–1.44)	< 0.001

ORs and RRs are unadjusted estimates calculated from the refracture and non-refracture groups. *OR* odds ratio, *RR* relative risk, *CI* confidence interval

Female sex (OR, 1.46; 95% CI, 1.21–1.76; *P* < 0.001), hypertension or cardiac disease (OR, 1.36; 95% CI, 1.13–1.63; *P* = 0.001), visual impairment (OR, 1.52; 95% CI, 1.24–1.86; *P* < 0.001), and a history of syncope (OR, 1.38; 95% CI, 1.06–1.80; *P* = 0.019) were also associated with higher refracture occurrence. Cerebrovascular disease showed only a borderline association (OR, 1.27; 95% CI, 1.01–1.59; *P* = 0.051).

Postoperative factors showed similar crude patterns. Non-standard rehabilitation was associated with higher refracture occurrence (OR, 1.58; 95% CI, 1.32–1.89; RR, 1.36; 95% CI, 1.21–1.52; *P* < 0.001), as was a Harris hip score < 80 (OR, 1.52; 95% CI, 1.28–1.82; RR, 1.30; 95% CI, 1.17–1.44; *P* < 0.001). Index fracture type was not associated with refracture (intertrochanteric vs femoral neck fracture: OR, 1.08; 95% CI, 0.91–1.28; *P* = 0.415).

A Cox proportional hazards model was additionally fitted to account for the time-to-event nature of refracture and was subsequently included as the conventional statistical comparator in the predictive analyses.

### Performance of machine learning models

**Fig. 3:**
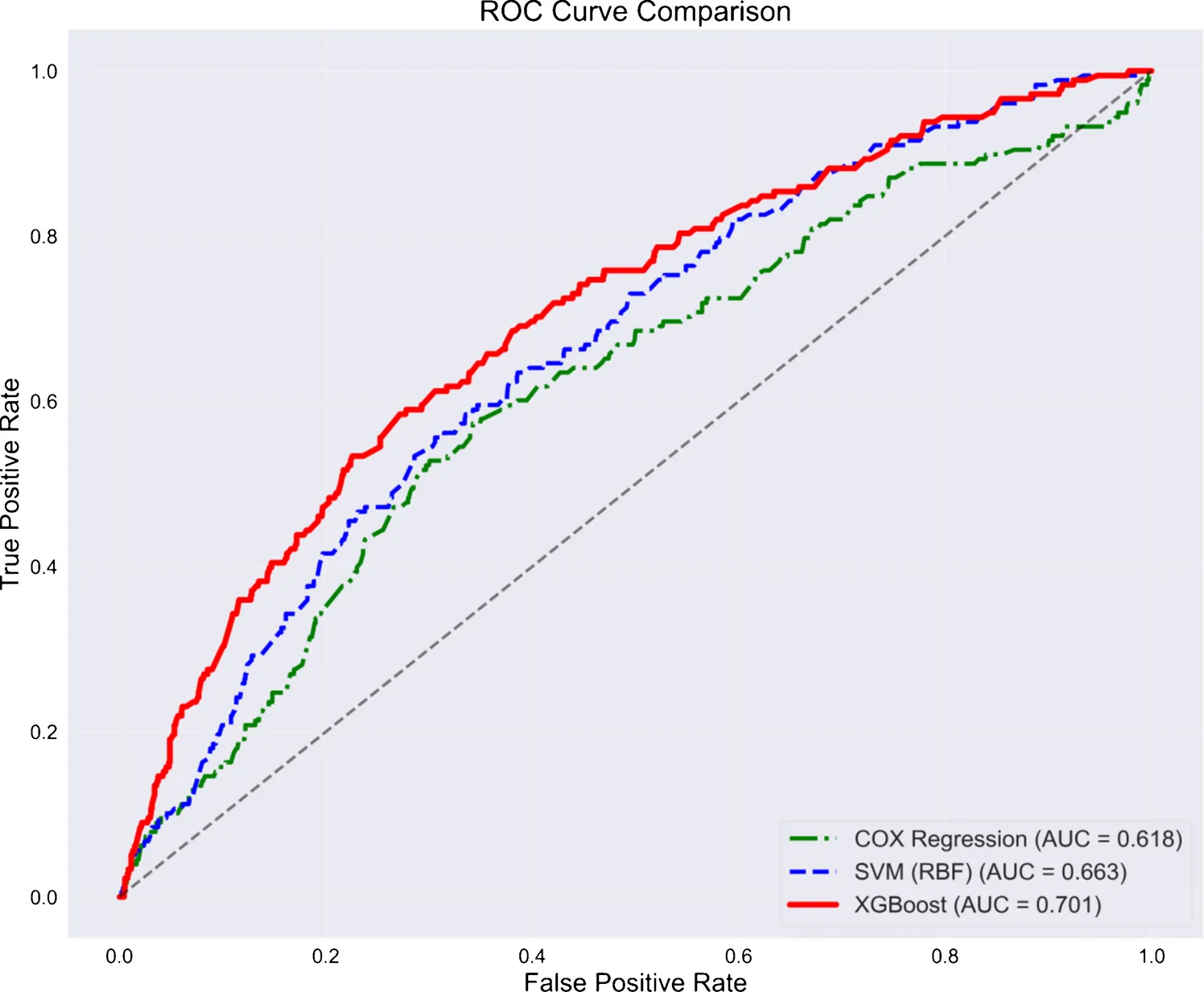
Receiver operating characteristic curves comparing XGBoost, RBF-SVM, and Cox regression in the held-out test set

We developed supervised binary classifiers to predict three year refracture using routinely available perioperative variables (age, sex, BMI category, osteoporosis, major comorbidities, visual impairment, syncope history, index fracture type, rehabilitation status, and postoperative Harris hip score). From the primary cohort (*n* = 4,418; refracture *n* = 594), we randomly split the data into a training set (70%; *n* = 3,092) and an independent test set (30%; *n* = 1,326). XGBoost demonstrated the highest discrimination among the evaluated approaches in the test set, with an AUC of 0.701 (95% CI, 0.657–0.743), compared with 0.663 (95% CI, 0.619–0.703) for SVM and 0.618 (95% CI, 0.570–0.660) for Cox regression (Fig. 3). At the default classification threshold of 0.50, XGBoost achieved an accuracy of 66.5%, sensitivity of 61.8%, specificity of 67.2%, and F1 score of 0.331. SVM achieved an accuracy of 63.0%, sensitivity of 59.6%, specificity of 63.6%, and F1 score of 0.302. Based on the confusion matrix, Cox regression yielded an accuracy of 57.9%, sensitivity of 63.5%, specificity of 57.1%, and F1 score of 0.288. Thus, although Cox regression showed slightly higher sensitivity than the other models at the evaluated cutoff, its overall discrimination and specificity were lower (Table 3).

**Table 3:** Predictive performance of the evaluated models in the test set

Model	AUC (95% CI)	Accuracy	Sensitivity	Specificity	F1 score
Cox Regression	0.618 (0.570–0.660)	57.9%	63.5%	57.1%	0.288
SVM XGBoost	0.663 (0.619–0.703) 0.701 (0.657–0.743)	63.0% 66.5%	59.6% 61.8%	63.6% 67.2%	0.302 0.331

*SVM* support vector machine, *XGBoost* extreme gradient boosting

Decision curve analysis further evaluated the potential clinical utility of the three prediction approaches (Figure S2). XGBoost provided the highest net benefit across threshold probabilities of approximately 7%–32%, outperforming the treat-all and treat-none strategies as well as SVM and Cox regression throughout this range. The net benefit of XGBoost remained non-negative up to approximately 35%. In comparison, SVM and Cox regression showed narrower ranges of positive net benefit, approximately 7%–25% and 7%–20%, respectively. These findings indicate that XGBoost provided the broadest range of potential decision-analytic benefit among the evaluated approaches, despite its moderate overall discrimination.

**Fig. 4:**
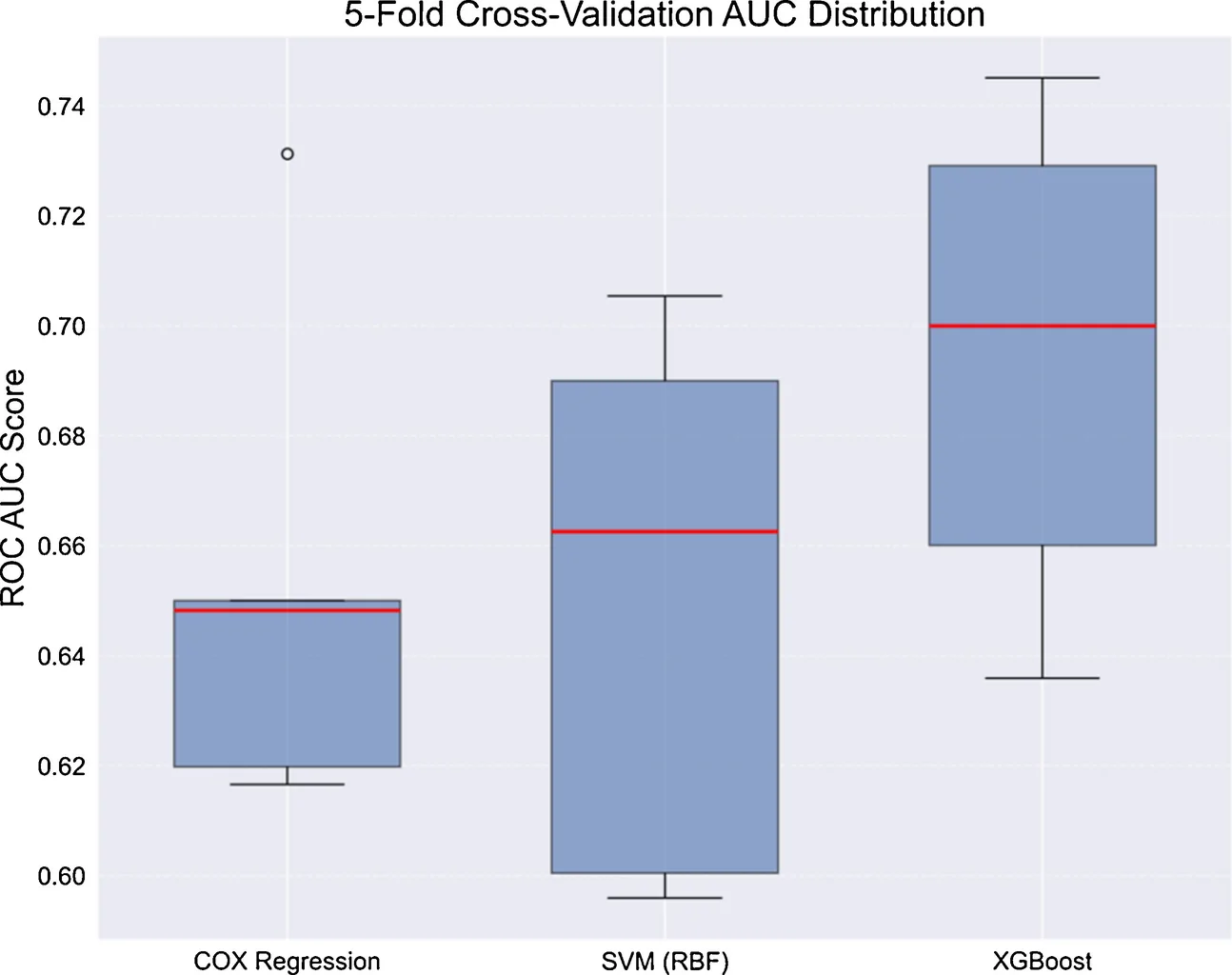
Model stability analysis using 5-Fold cross-validation

**Fig. 5:**
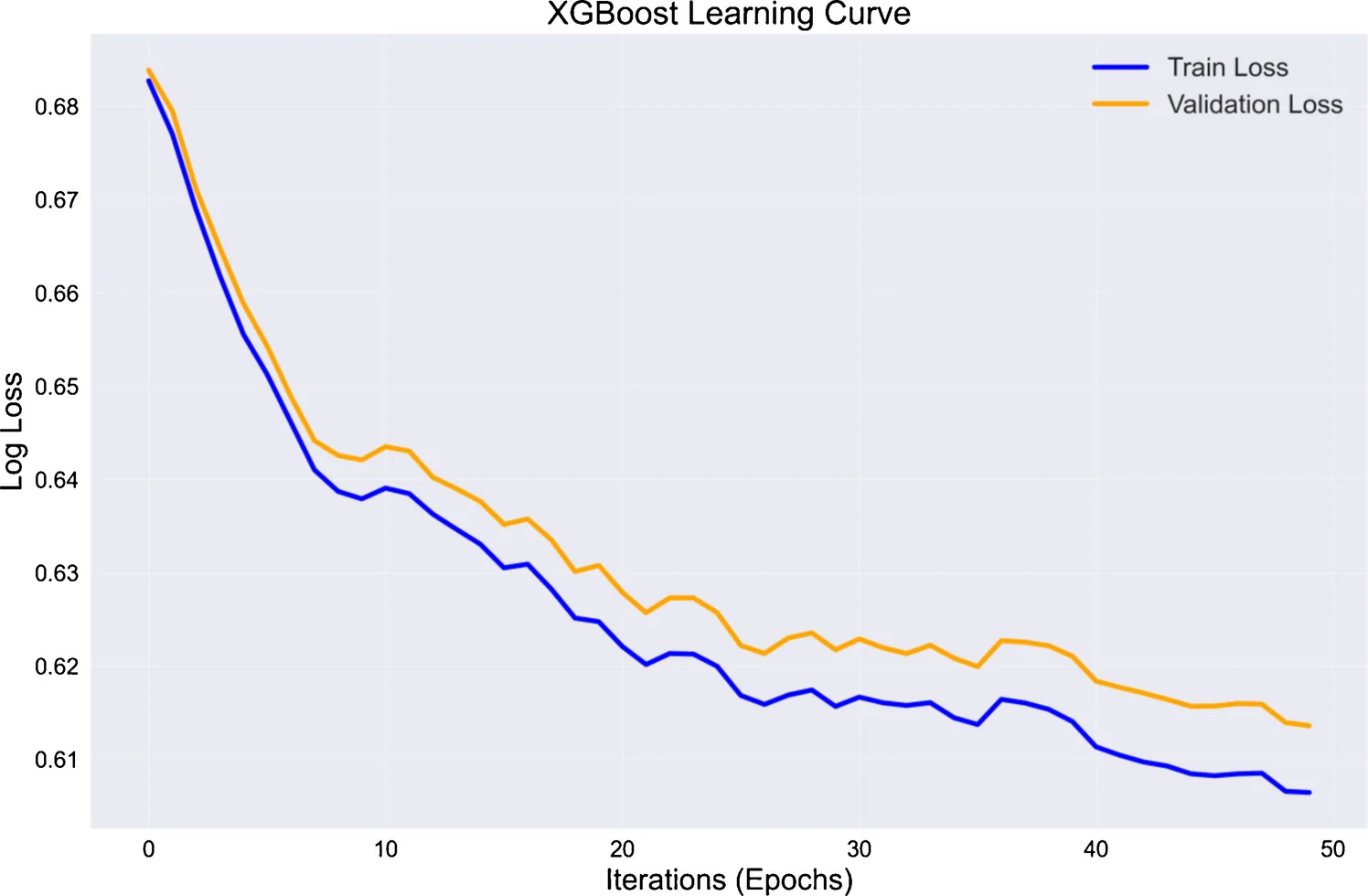
XGBoost training and validation loss trajectory

**Fig. 6:**
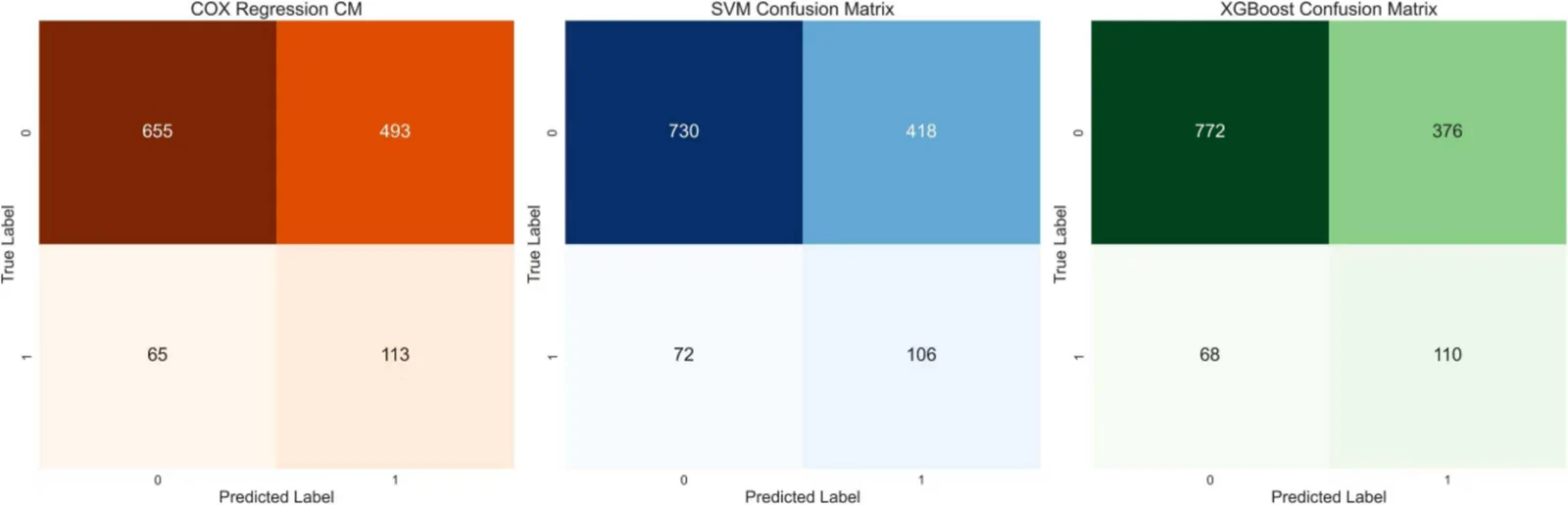
Confusion matrices for Cox regression, SVM, and XGBoost in the held-out test set at the default classification threshold

Five-fold cross-validation showed that XGBoost generally achieved higher AUC values and a higher central AUC distribution than SVM and Cox regression across folds (Fig. 4), indicating higher cross-validated discrimination for XGBoost within the development dataset. The learning curves for XGBoost showed progressive reductions in training and validation log loss across boosting iterations, without marked divergence between the two curves, providing no evidence of severe overfitting within the development dataset (Fig. 5). Confusion matrices in the held-out test set further illustrated differences in classification behaviour among the three models (Fig. 6). XGBoost correctly classified 772 of 1,148 non-refracture cases and 110 of 178 refracture cases at the default threshold, whereas SVM correctly classified 730 and 106, respectively. Cox regression correctly classified 655 non-refracture cases and 113 refracture cases, reflecting its somewhat higher sensitivity but substantially lower specificity.

### Feature importance and explainability

**Fig. 7:**
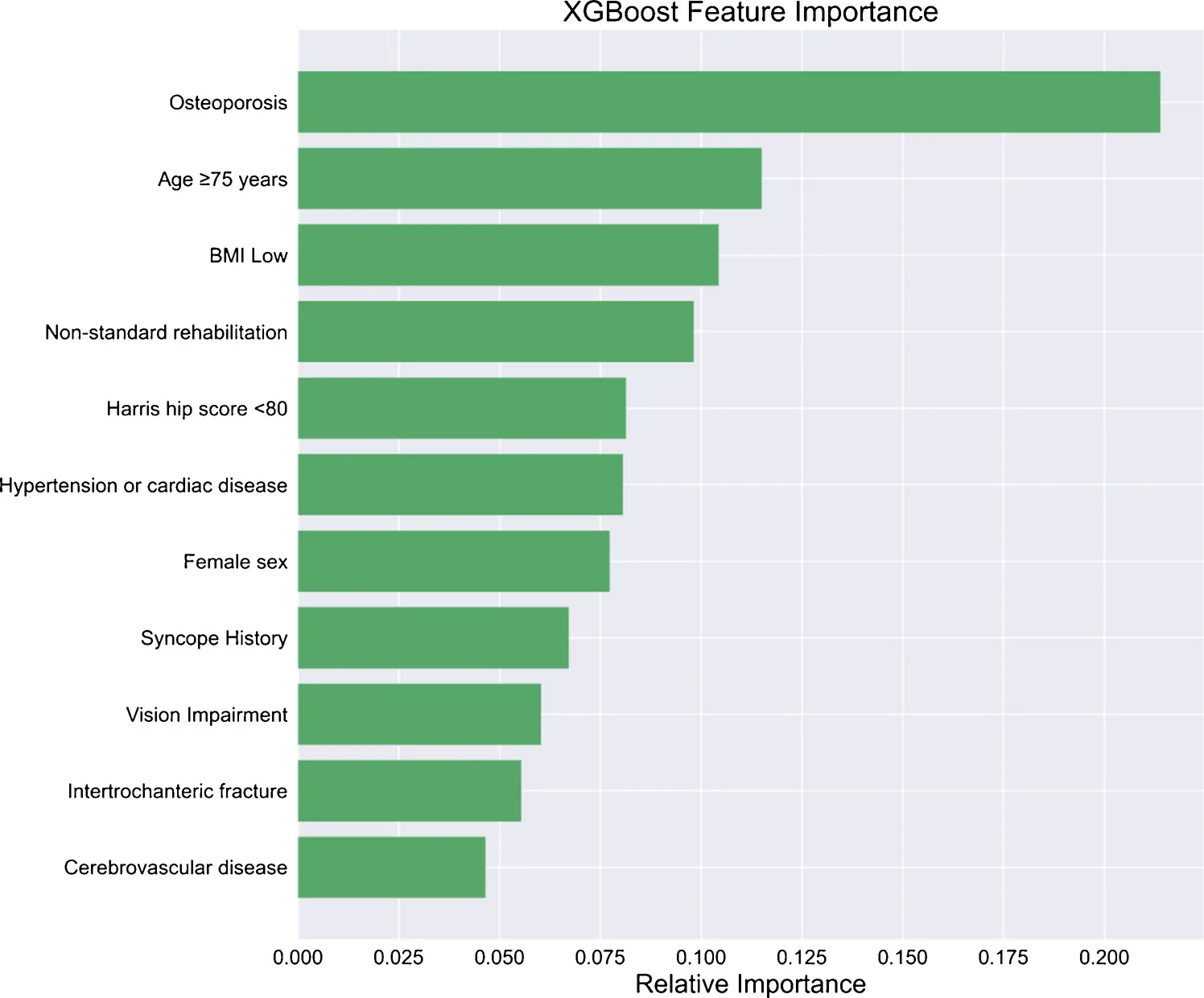
Relative feature importance ranking in the XGBoost model

Global feature-importance analysis was performed to characterize the relative contribution of individual predictors to the XGBoost model. Osteoporosis had the highest feature-importance score, followed by age ≥ 75 years, BMI < 18.5 kg/m^2^, non-standard rehabilitation, and a postoperative Harris hip score < 80. Female sex, hypertension or cardiac disease, visual impairment, history of syncope, index fracture type, and cerebrovascular disease contributed to the model to a lesser extent (Fig. 7). The prominence of osteoporosis, advanced age, low BMI, rehabilitation status, and postoperative function was broadly consistent with the patterns observed in the crude association analyses. However, feature-importance scores reflect contribution to model prediction and should not be interpreted as adjusted effect estimates or evidence of causal importance.

## Discussion

In this large real-world cohort of 4,418 older adults undergoing surgery for a first fragility hip fracture, 594 patients (13.4%) sustained a secondary fragility fracture within three years. The principal finding was that refracture risk reflected a multidimensional interaction of skeletal fragility, fall-related vulnerability, and postoperative recovery. Osteoporosis, advanced age, low BMI, female sex, visual impairment, syncope, cardiometabolic comorbidity, non-standard rehabilitation, and poorer postoperative hip function were associated with higher crude refracture occurrence, whereas index fracture type was not. Among patients who sustained refracture, stratified time-to-refracture analyses further suggested differences in event timing according to osteoporosis status, rehabilitation status, and postoperative Harris hip score. These findings support a comprehensive secondary-prevention strategy that integrates bone health, fall risk, and functional recovery.

The strong association between osteoporosis, advanced age, and low BMI is consistent with the established contribution of skeletal fragility and reduced physiological reserve to recurrent fracture. However, the associations of visual impairment and syncope highlight the importance of non-skeletal contributors to injurious falls [24, 25]. Assessment of vision, syncope or orthostatic symptoms, gait stability, and fall susceptibility may therefore complement osteoporosis management in postoperative care [26, 27]. Rehabilitation also emerged as an important clinical domain. Patients receiving non-standard rehabilitation or having a Harris hip score < 80 experienced more refractures, and among patients who sustained refracture, these factors were also associated with differences in the timing of refracture. Because this was an observational study, these associations do not establish causality and may partly reflect frailty, comorbidity, social support, motivation, or access to care. Nevertheless, structured rehabilitation incorporating strength, balance, gait, and safe mobility may represent an actionable component of comprehensive secondary fracture prevention [19, 28].

We further evaluated whether routinely available multidimensional clinical information could support postoperative risk stratification. XGBoost achieved better discrimination than SVM and Cox regression, although its AUC of 0.701 indicates moderate discrimination. At the default threshold of 0.50, sensitivity was 61.8% and specificity was 67.2%, indicating that the model should not be used as a standalone diagnostic or treatment-decision instrument. Its more appropriate role is screening and triage, identifying patients who may benefit from more intensive assessment [29]. Decision curve analysis provided additional evidence of potential clinical utility. XGBoost showed the greatest net benefit across threshold probabilities of approximately 7%–32%, outperforming treat-all, treat-none, SVM, and Cox regression strategies over this range, with non-negative net benefit extending to approximately 35%. These findings suggest that moderate discrimination can still translate into potentially useful decision support when the model is applied to an appropriate clinical decision. However, decision curve analysis estimates potential net benefit under specified threshold preferences and does not demonstrate improved patient outcomes. Prospective clinical-impact studies are therefore required before routine implementation.

The model should also be considered alongside established fracture-risk tools such as FRAX. A direct comparison was not possible because FRAX probabilities were not systematically available. Moreover, the two approaches address different clinical contexts. FRAX estimates longer-term osteoporotic fracture probability using established clinical risk factors, whereas the present model was developed specifically after fragility hip fracture surgery and incorporated postoperative rehabilitation and functional variables. XGBoost should therefore be viewed as a complementary triage tool, not a substitute for FRAX, osteoporosis assessment, or clinical judgement. Future studies combining conventional fracture-risk measures with postoperative functional and fall-related variables may provide more comprehensive risk stratification [19].

Our findings should also be interpreted in the context of previous prediction studies. Liang et al. developed a nomogram for contralateral hip fracture in 734 patients and reported high discrimination [30], while Shimizu et al. reported moderate performance using machine-learning approaches in a heterogeneous surgically treated fragility-fracture population [19]. Wu et al. subsequently reported excellent performance using AutoGluon in older patients with hip fracture [31]. Kim et al. used CT-based deep learning and achieved AUCs of approximately 0.73–0.74 for subsequent fracture prediction [16]. Differences in cohort composition, outcome definition, prediction horizon, available predictors, preprocessing, and validation strategy make direct comparisons of AUC inappropriate. Importantly, imaging-based models may capture skeletal architecture and bone quality that are incompletely represented by routine clinical variables, whereas our model incorporated rehabilitation, functional recovery, and fall-related factors. Future multimodal models integrating BMD or quantitative imaging with clinical and functional information may therefore offer greater predictive value [32, 33].

Feature-importance analysis identified osteoporosis, advanced age, low BMI, non-standard rehabilitation, and poorer postoperative hip function among the most influential predictors. This pattern was broadly consistent with the crude analyses and highlighted the coexistence of relatively fixed factors and potentially modifiable postoperative characteristics. Feature importance, however, reflects contribution to prediction and does not establish causality or provide an adjusted effect estimate. Its primary value is therefore to demonstrate that the model relies on clinically plausible risk domains.

This study has several strengths. The cohort was relatively large, included 4,418 patients and 594 refracture events, and captured secondary fragility fractures across multiple major osteoporotic sites. The analysis also incorporated postoperative rehabilitation and functional recovery, which are potentially modifiable and less consistently represented in previous prediction models. In addition to AUC, we assessed classification performance, internal cross-validation, and decision-analytic net benefit, providing a more comprehensive evaluation of potential clinical utility.

Several limitations warrant consideration. First, the single-centre retrospective design may limit generalizability because patient characteristics, rehabilitation access, and osteoporosis management may differ across healthcare settings. Second, selection and survivor bias are possible, as 818 eligible patients who died or were lost to follow-up before three years were excluded, potentially underestimating refracture burden among the frailest patients. Third, several established risk factors were incompletely captured, including BMD T-scores, FRAX, recurrent falls, osteoporosis medication exposure, and vitamin D levels, which may have limited predictive performance and contributed to residual confounding. Finally, rehabilitation status and Harris hip score represent postoperative information, so the model should not be considered an admission-only prediction tool. Formal calibration assessment was not included in the present analysis, which limits interpretation of the agreement between predicted and observed absolute risks. Calibration, including assessment of calibration slope and intercept, should therefore be evaluated during subsequent temporal and external validation. Future studies should use standardized prediction landmarks, incorporate more comprehensive clinical and treatment data, and undergo temporal and geographic external validation before clinical implementation.

## Conclusion

In this large real-world cohort of older adults undergoing surgery for a first fragility hip fracture, secondary fragility fractures were common and associated with a combination of skeletal fragility, fall-related vulnerability, and suboptimal postoperative recovery. The XGBoost model provided moderate discrimination but demonstrated a favourable range of decision-analytic net benefit compared with conventional comparators. These findings support its further evaluation as an adjunctive triage tool for identifying patients who may benefit from intensified secondary prevention, rather than as a standalone diagnostic model. External validation and prospective clinical-impact testing are required before routine implementation.

## Data Availability

No datasets were generated or analysed during the current study.
